# Epitope-Specific Serological Assays for RSV: Conformation Matters

**DOI:** 10.3390/vaccines7010023

**Published:** 2019-02-23

**Authors:** Emily Phung, Lauren A. Chang, Kaitlyn M. Morabito, Masaru Kanekiyo, Man Chen, Deepika Nair, Azad Kumar, Grace L. Chen, Julie E. Ledgerwood, Barney S. Graham, Tracy J. Ruckwardt

**Affiliations:** 1 Vaccine Research Center, National Institutes of Allergy and Infectious Diseases, National Institutes of Health, Bethesda, MD 20892, USA; emily.phung@nih.gov (E.P.); lauren.chang@nih.gov (L.A.C.); kaitlyn.dambach@nih.gov (K.M.M.); masaru.kanekiyo@nih.gov (M.K.); manchen@mail.nih.gov (M.C.); deepika.nair@nih.gov (D.N.); azad.kumar@nih.gov (A.K.); grace.chen@nih.gov (G.L.C.); JUMARTIN@niaid.nih.gov (J.E.L.); bgraham@nih.gov (B.S.G.); 2 Institute for Biomedical Sciences, George Washington University, Washington, DC 20052, USA

**Keywords:** respiratory syncytial virus, monoclonal antibody competition, palivizumab competition assay, protein conformation, ELISA, immune correlates of protection

## Abstract

Respiratory syncytial virus (RSV) causes substantial morbidity and mortality in children and older adults. An effective vaccine must elicit neutralizing antibodies targeting the RSV fusion (F) protein, which exists in two major conformations, pre-fusion (pre-F) and post-fusion (post-F). Although 50% of the surface is shared, pre-F contains highly neutralization-sensitive antigenic sites not present on post-F. Recent advancement of several subunit F-based vaccine trials has spurred interest in quantifying and understanding the protective potential of antibodies directed to individual antigenic sites. Monoclonal antibody competition ELISAs are being used to measure these endpoints, but the impact of F conformation and competition from antibodies binding to adjacent antigenic sites has not been thoroughly investigated. Since this information is critical for interpreting clinical trial outcomes and defining serological correlates of protection, we optimized assays to evaluate D25-competing antibodies (DCA) to antigenic site Ø on pre-F, and compared readouts of palivizumab-competing antibodies (PCA) to site II on both pre-F and post-F. We show that antibodies to adjacent antigenic sites can contribute to DCA and PCA readouts, and that cross-competition from non-targeted sites is especially confounding when PCA is measured using a post-F substrate. While measuring DCA and PCA levels may be useful to delineate the role of antibodies targeting the apex and side of the F protein, respectively, the assay limitations and caveats should be considered when conducting immune monitoring during vaccine trials and defining correlates of protection.

## 1. Introduction

Respiratory syncytial virus (RSV) can cause severe acute lower respiratory infections (ALRI), resulting in 55,000 to 199,000 deaths globally per year in children under the age of five [1]. In adults age 65 years or older, RSV proves to be a substantial health burden responsible for 8% of ALRI-related deaths in US hospitals [2]. Despite five decades of vaccine development effort, no RSV vaccine is available. The primary goal of an RSV vaccine is to prevent severe disease in those at greatest risk—infants under 6 months of age and older adults [3]. Many vaccines tested in these populations have proven unsuccessful, revealing challenges and raising concerns for developing an RSV vaccine for these vulnerable populations [4,5,6]. In addition, the lack of a definitive correlate of protection against infection or severe disease complicates efforts to evaluate vaccine efficacy without including large numbers of subjects in late-phase clinical trials [7]. Despite the complicated history and challenges of RSV vaccination, there are numerous promising vaccine candidates under clinical evaluation that either directly target infants and the elderly or aim to protect infants through immunization of pregnant women or older children [8,9,10]. Defining serological readouts that best correlate with protection from disease will be a critical advance toward developing and advancing the most efficacious RSV vaccines.

RSV neutralizing activity has often been correlated with protection in infants, and antibody alone is capable of mediating protection from severe disease [7]. While the envelope of RSV contains two major surface glycoproteins (the fusion protein F and the attachment glycoprotein G), the relative conservation of the F protein makes it an attractive target for active vaccination and passive antibody administration [11]. Palivizumab (Synagis), a monoclonal antibody (mAb) that recognizes the F protein, has significantly reduced hospitalization in high-risk infants when administered as monthly prophylaxis [12,13]. There are two major conformations of the F protein, the active pre-fusion form (pre-F) and the post-fusion form (post-F) adopted after triggering [14,15,16]. Antigenic sites on the F protein have been defined, first through mAb competition and structural studies of F-mAb complexes followed by dividing the surface of pre-F into six non-overlapping structural domains [17,18,19,20]. The side of post-F displays four antigenic sites designated as antigenic sites I, II, III, and IV [18]. Unique antigenic sites on the apex of pre-F, designated sites Ø and V, bind antibodies with substantially better neutralization potency than site II-directed palivizumab [21,22,23,24,25,26]. In addition to displaying the pre-F exclusive sites Ø and V, the side of pre-F has similar structural topology to post-F, retaining antigenic sites I-IV. While antibodies that target sites II and IV bind similarly to pre-F and post-F, antibodies to sites III and I preferentially bind pre-F and post-F, respectively [18]. Determination of the pre-F structure has informed advances in passive immunoprophylaxis and a site Ø directed antibody, D25, is being clinically evaluated with half-life extending mutations as a replacement for palivizumab [27]. Additionally, stabilizing mutations have been introduced into the F protein to preserve the major viral sites of vulnerability, offering an optimal antigen for active vaccination [28]. As F-based vaccine candidates continue in clinical evaluation, it has become increasingly important to consider immunological readouts and what they tell us about both natural and vaccine-elicited immunity to RSV.

Neutralization assays have long been the gold standard for measuring functional antibody responses following vaccination. Similarly, standard ELISA assays are used to demonstrate an increase in F-binding antibodies. However, these two readouts have provided discordant results following vaccination with post-F antigens. In several trials, the increase in binding antibody far exceeded the improvement in neutralizing activity [29,30], indicating that while immunogenic, post-F vaccines elicit primarily non- or weakly- neutralizing antibodies. Both the neutralization assay and ELISA measure responses to F as a whole and lack the refinement necessary to distinguish antibody specificity to different antigenic sites or preference for pre-or post-F conformation. Based on the efficacy of palivizumab administered to high-risk infants, assays have also been developed to measure the induction of responses that compete with palivizumab for binding to the F protein. Because palivizumab can protect from disease, it is logical to assume that antibodies that compete with palivizumab should have protective capabilities, and therefore, palivizumab-competing antibody (PCA) concentrations in serum could serve as a surrogate for neutralization. As a result, readouts of PCA levels in serum are routinely included as a measure of vaccine-elicited immunity. However, most of these PCA levels have been measured using a post-F or structurally-undefined antigen [31,32,33,34,35]. Given our current understanding of the biology of the F protein and the advancement of several pre-F vaccine candidates, it is likely that this is an inadequate and potentially misleading immunological readout.

While the PCA measured using post-F or structurally-undefined F protein has become a standard immunological readout for vaccine studies, its utility for pre-F vaccines or as a correlate of protection has yet to be determined. Both pre-F and post-F contain the palivizumab-binding helix-turn-helix epitope within antigenic site II [14,15,36], but it is unclear to what extent the relationship with adjacent antigenic sites and presentation context of this motif affect the readout of a PCA assay. Additionally, whether pre-F or post-F is used as a substrate, the PCA assay fails to provide any information about antibody responses to the neutralization-sensitive antigenic sites Ø and V present exclusively on pre-F, which is an important immunological readout for pre-F-based vaccine trials. To address these limitations, we sought to (1) evaluate the impact of protein conformation on PCA readouts and (2) develop an assay to quantify D25-competing antibody (DCA) levels in polyclonal sera. We optimized three ELISA assays to measure serum levels of pre-F DCA, pre-F PCA, and post-F PCA. We then used a panel of site-specific mAbs to evaluate the contribution of antibodies targeting adjacent sites to the readouts from each assay. Finally, we measured mAb-competing antibody levels in non-human primates (NHP) immunized with different conformations of the F protein to illustrate how immune history complicates interpretation of mAb competition assays. Our comprehensive approach to dissecting the impact of F conformation in mAb competition assays implicates protein conformation as a critical determinant of assay readouts and provides a basis to improve the assessment of RSV F vaccines.

## 2. Materials and Methods 

### 2.1. Human Sera

Sera from 58 healthy subjects between the ages of 18 and 65 were obtained with informed consent from subjects enrolled in the observational study VRC 200, A Multicenter Specimen Collection Protocol to Obtain Human Biological Samples for Research Studies (ClinicalTrials.gov identifier: NCT00067054). Subjects were randomly selected for testing with no restrictions on age or gender.

### 2.2. Protein Expression and Characterization

Pre-F and post-F proteins were produced from previously characterized constructs of DS-Cav1 [28] and RSV FdFP [15] by transient transfection of Expi293F cells. Expressed proteins contained a C-terminal hexa-histidine tag and Strep-tag for purification by affinity chromatography over Ni-nitrilotriacetic acid (NTA) (GE Healthcare, Pittsburgh, PA, USA) and Strep-Tactin resin (IBA Lifesciences, Göttingen, Germany) followed by a fast protein liquid chromatography superose column (Bio-Rad, Richmond, CA, USA) to isolate trimeric protein. All F proteins were tested for antigenicity and conformation by assessing binding to a panel of monoclonal antibodies targeting known antigenic sites on pre-F or post-F (D25 and 5C4: site Ø, Motavizumab: site II, AM14: quaternary).

### 2.3. Expression and Purification of Antibodies and Antigen Binding Fragments (Fabs)

Antibodies were expressed by transfection of heavy and light chain plasmids together into Expi293F cells grown in suspension at 37 °C. Six days later, culture supernatants were harvested and passed through Protein A agarose (ThermoFisher, Atlanta, GA, USA), and bound antibodies were washed with PBS prior to elution with IgG elution buffer (ThermoFisher) into 1 M Tris-HCl (pH 8.0). Fabs were made by digesting antibodies overnight with Lys-C (New England Biolabs, Beverly, MA, USA) or Papain (Pierce Fab Preparation Kit, ThermoFisher) following manufacturer’s instructions. Fc fragments were removed with Protein A (ThermoFisher) or Protein G Sepharose Fast Flow (GE Healthcare) agarose, and successful digestion of antibodies was confirmed using 4–12% Bis-Tris SDS-Page gels (Invitrogen, Carlsbad, CA, USA).

### 2.4. Monoclonal Antibody Competition ELISA

Competition ELISA assays were optimized for coating conditions and biotinylated antibody concentrations to maximize their dynamic range and keep similar coating concentrations for the two PCA assays. D25 and palivizumab were biotinylated with an EZ-Link-Sulfo-NHS-LC-biotinylation kit (ThermoFisher) following manufacturer’s instructions. Coating and biotinylated antibody concentrations selected for each assay, respectively, were: 4 μg/mL and 2 μg/mL for the pre-F DCA assay, 500 ng/mL and 2 μg/mL for the pre-F PCA assay, 250 ng/mL and 100 ng/mL for the post-F PCA assay. Internal polyclonal serum controls were included in every run to check for consistency between assays. Plates were coated overnight at 4 °C with F protein in PBS. The following morning, 200 μL of 5% milk + PBS were used to block the plates for 1 h. In between each step, plates were washed 3× with PBS-T. Two-fold serial dilutions of sera (50 μL, starting at a 1:5 dilution) were performed in duplicate and added to the ELISA plate for a 15-min incubation before adding 50 μL of biotinylated monoclonal antibody and an additional one-hour incubation. Standard curves were generated using unbiotinylated D25 or palivizumab for each competition assay (2- fold dilutions starting at 25 μg/mL for pre-F DCA assay, 200 μg/mL for pre-F PCA assay, and 25 μg/mL for the post-F PCA assay). After washing, 100 μL of Streptavidin-HRP (1:2000; ThermoFisher) was added to the plates and incubated for one hour. The plates were developed using 100 μL of KPL SureBlue (SeraCare, Milford, MA, USA) for 7 min, and the reaction was stopped with 100 μL of sulfuric acid (0.501M). Plates were read at 450 nm and 650 nm on a SpectraMax Paradigm plate reader. For analysis, a 4-parameter nonlinear regression was performed to determine the concentration of unbiotinylated D25 or palivizumab monoclonal antibody necessary to achieve IC_50_. The concentrations of D25- and palivizumab- competing antibody within experimental samples were interpolated from the absorbance value at which experimental samples achieved 50% inhibition. The lower limit of quantitation was 4 μg/mL for the pre-F DCA assay, 24 μg/mL for the pre-F PCA assay, and 5 μg/mL for the post-F PCA assay. Sera with unmeasurable levels of competing antibody were assigned a value half the lower limit of quantitation.

### 2.5. Neutralization Assays

Neutralization assays were performed as previously described [37]. Briefly, HEp-2 cells were seeded at a density of 2.4 × 10^4^ cells/well in 384-well black optical bottom plates from ThermoFisher (Cat#142761). Serial 2-fold dilutions were performed on samples, either NHP sera or monoclonal antibodies, in a final volume of 40 µL. The starting dilution was 1:10 for sera and 1:10, 1:200, or 1:500 for the monoclonal antibodies. An equal volume of recombinant mKate-RSV subtype A (strain A2) was added to the diluted samples and incubated at 37 °C for 1 h [38]. After incubation, 50 µL of each diluted sample-virus mixture was added to the HEp-2 cells and incubated for 37 °C for 24 h. Fluorescence endpoints were recorded at 24 h using excitation at 588 nm and emission at 635 nm (SpectraMax M2e, Molecular Devices, San Jose, CA, USA). The IC_50_ titer for each sample was determined using a four-parameter non-linear regression curve fit with GraphPad Prism version 7 (GraphPad Software Inc., San Diego, CA, USA), which was then used to calculate the IC_50_ concentration of each monoclonal antibody or NHP sera.

### 2.6. K_D_ Determination

Avi-tagged pre-F and post-F (purified as described above) were biotinylated using the BirA Biotin-Protein Ligase Standard Reaction Kit (Avidity, Aurora, CO, USA). Biotinylation and conformation were confirmed using a FortéBio Octet HTX instrument (Molecular Devices, Fremont, CA, USA) by immobilizing biotinylated pre-F and post-F on streptavidin (SA) sensors and testing their binding to a panel of site-specific monoclonal RSV F antibodies (D25 and 5C4: site Ø, ADI-15640: site I, Motavizumab: site II, MPE8: site III, ADI-15569: site V, AM14: quaternary). All biosensors were hydrated in PBS prior to use, and pre-F and post-F proteins were immobilized on SA biosensors through conjugated biotin. After briefly dipping in assay buffer (1% BSA in PBS), the biosensors were dipped in a 3-fold dilution series of Fab for 5 min. Biosensors were then dipped in assay buffer to allow Fab to dissociate from F for 10 min. All assay steps were performed at 30 °C with agitation set at 1000 rpm in the Octet HTX instrument (Molecular Devices). Correction to subtract non-specific baseline drift was carried out by subtracting the measurements recorded for a bare sensor. Data analysis and curve fitting were carried out using Octet analysis software (version 9.0). Experimental data were fitted with the binding equations describing a 1:1 interaction. Global analyses of the complete data sets assuming binding was reversible (full dissociation) were carried out using nonlinear least-squares fitting, allowing a single set of binding parameters to be obtained simultaneously for all concentrations used in each experiment. Fabs with a maximum response unit of less than 0.1 nm were excluded from *K*_D_ calculation. 

### 2.7. NHP Immunizations

All animal experiments were reviewed and approved by the Animal Care and Use Committee of the Vaccine Research Center, NIAID, NIH, and all animals were housed and cared for in accordance with local, state, federal, and institute policies in an American Association for Accreditation of Laboratory Animal Care (AAALAC)-accredited facility at the NIH. *Macaca mulatta* animals of Indian origin weighing 8.76–14.68 kg were intramuscularly injected with immunogens at week 0, week 4, and week 26. The frozen RSV F variant immunogen proteins were thawed on ice and mixed with poly I:C with 50 µg/animal injections taking place within one hour of immunogen: adjuvant preparation.

### 2.8. Statistical Analysis

Correlation between pre-F and post-F PCA levels in addition to DCA and PCA readouts to neutralization were determined by Spearman correlation analysis. All *t*-tests comparisons of non-human primate DCA, PCA, and neutralization readouts were done using Microsoft Excel.

## 3. Results

### 3.1. Optimization of mAb Competition Assays to Measure Site-specific Antibody Responses

**Figure 1:**
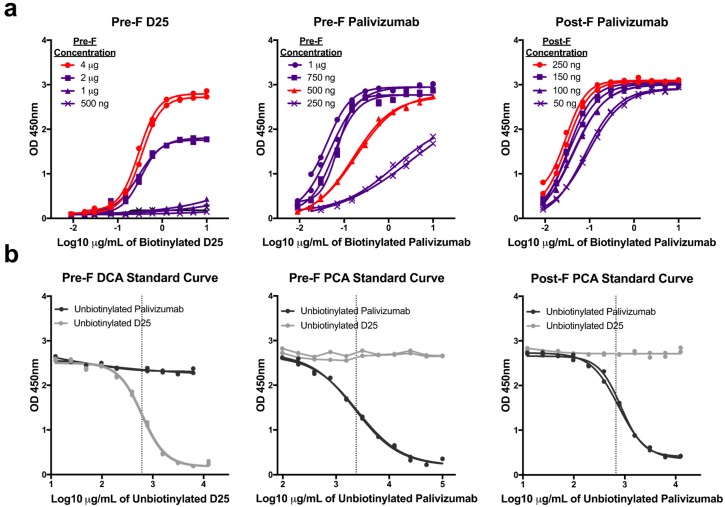
Development of monoclonal antibody (mAb) competition assays. (**a**) Serial dilutions of biotinylated D25 or palivizumab detection antibodies were tested for binding to pre-fusion (pre-F) or post-fusion (post-F) coated overnight on plates at the indicated concentrations. The samples highlighted in red indicate the coating concentration that was selected for each assay. Sub-saturating concentrations of detection antibodies were determined, and final assay conditions selected for each assay were: pre-F DCA: 4 µg coating, 2 µg/mL D25, pre-F PCA: 500 ng coating, 2 µg/mL palivizumab, post-F PCA: 250 ng coating, 0.1 µg/mL palivizumab. Lines with identical symbols indicate technical replicates. (**b**) Serial dilutions of unbiotinylated D25 and palivizumab were used in each assay to generate a full standard curve and determine the IC_50_ of antibody identical to the detection reagent (dotted line). The concentrations of D25-competing antibody (DCA) and palivizumab-competing antibody (PCA) within experimental samples were then interpolated from the absorbance value at which experimental samples achieved 50% inhibition. Lines with identical symbols are technical replicates.

Since the PCA assay has not been standardized across laboratories, we independently developed three mAb competition assays by optimizing the concentration of antigen coated on the plate and of the biotinylated detection antibody. First, we coated plates with increasing concentrations of purified pre-F or post-F and performed serial two-fold dilutions of biotinylated D25 (pre-F only) and palivizumab (both pre-F and post-F) antibodies. Antigen coating concentrations that offered sufficient dynamic range were selected: 4 µg/mL for pre-F DCA assay, 500 ng/mL for pre-F PCA assay, 250 ng/mL for post-F PCA assay. Next, we determined concentrations of biotinylated mAb that achieved 90% saturation of the signal to be used in competition assays: 2 µg/mL for pre-F DCA and pre-F PCA assays and 0.1 µg/mL for post-F PCA assay (Figure 1a). Unbiotinylated D25 and palivizumab were then serially diluted to generate standard curves and determine the concentration of mAb necessary to achieve a 50% inhibition (IC_50_) of the signal (Figure 1b). While the IC_50_ of the unbiotinylated form of the competing antibody between runs is generally consistent for each competition assay, it was necessary to include this control to establish the IC_50_ for each run. The IC_50_ provided by unbiotinylated mAb generated with each run was then used to interpolate µg/mL of DCA or PCA in experimental samples. 

### 3.2. Quantifying Site-Specific Antibody Concentrations in Healthy Human Sera

**Figure 2:**
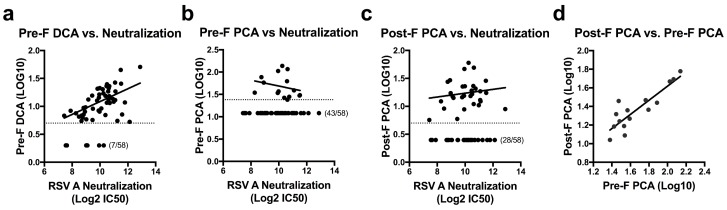
Quantitation of site-specific antibody concentrations and their correlation to neutralization in healthy human sera. (**a–c**) Pre-F DCA (*r* = 0.487, *p* = 0.0003) (**a**), pre-F PCA (*r* = −0.335, *p* = 0.221) (**b**), and post-F PCA (*r* = 0.126, *p* = 0.508) (**c**) levels were quantified and plotted against neutralization titers in 58 healthy human subjects. Dotted lines indicate the limit of quantitation for each assay (pre-F DCA: 4 µg/mL, pre-F PCA: 24 µg/mL, post-F PCA: 5 µg/mL). Samples falling below the limit of quantitation were set at a value of half the limit of quantitation. Number of undetectable subjects are indicated. (**d**) Correlation of pre-F PCA and post-F PCA concentrations in the 15 subjects where both could be quantitated (*r* = 0.793, *p* = 0.0007). All results were reported as the average of technical duplicates for each sample.

To assess RSV F antibody levels in naturally infected humans, we determined the concentration of DCA and PCA for 58 randomly-selected healthy adults between the ages of 18–65. Serial dilutions of sera were added to plates coated with pre-F or post-F before the addition of the biotinylated D25 or palivizumab. The concentration of serum required to block 50% of biotinylated D25 or palivizumab was calculated and multiplied by the IC_50_ of unbiotinylated D25 or palivizumab to establish DCA and PCA levels for each sample. Based on an initial serum dilution of 1:10 and the IC_50_ of unbiotinylated mAb, the limit of quantitation was set as 4 µg/mL for the pre-F DCA assay, 24 µg/mL for the pre-F PCA assay, and 5 µg/mL for the post-F PCA assay. Samples falling below these limits of quantitation were assigned values of 2 µg/mL, 12 µg/mL, and 2.5 µg/mL, respectively. Measurements of pre-F PCA were limited by the relatively high IC_50_ of unbiotinylated palivizumab in this assay (average of 2.07 µg/mL over five runs, Figure 1b) and an inability to extrapolate quantitation beyond the original serum dilution. This restriction on quantitation allowed for determination of pre-F PCA in only 15 of the 58 subjects tested (26%), while pre-F DCA and post-F PCA levels could be determined for 88% and 52% of subjects, respectively. While the mAb competition assays did not directly correlate with neutralization activity in this set of normal human subjects, the pre-F DCA assay was the best predictor of neutralization (*r* = 0.487, *p* = 0.0003) (Figure 2a). The number of subjects below the limit of quantitation for the pre-F PCA and post-F PCA assays limited the ability to correlate these readouts to neutralization (Figure 2b,c). For subjects with measurable PCA in both the pre-F and post-F assay, the µg/mL readouts correlated well (*r* = 0.793, *p* = 0.0007, Figure 2d). These data suggest that for polyclonal sera from antigen-experienced adults, similar readouts are obtained when using RSV F in either of its conformations as a substrate for the mAb competition ELISA.

### 3.3. Antibodies to Adjacent Sites Contribute to Readouts of Mab Competing Antibody

The elucidation of major antigenic sites on RSV pre-F and the delineation of surfaces shared on pre-F and post-F have contributed to several studies mapping the serological response to RSV F in antigen-experienced adults and children [21,25,26,39] and infants after their first RSV infection [40,41,42,43]. Despite these efforts, the impact of RSV F conformation on mAb competition readouts has not been fully explored. To determine how structural differences between pre-F and post-F could alter the binding profiles of mAbs targeting the same antigenic site and assess how antibodies to adjacent sites contribute to mAb competition assay readouts, we expressed an investigational panel of 39 mAbs, selecting several antibodies with reported specificity to each of the six defined antigenic sites (Ø-V) with available published sequences [13,14,21,23,24,40,44,45,46,47]. After expression, the neutralizing activity (IC_50_) of each full-length Ig was determined using a fluorescence reporter-based neutralization assay (Table 1). We additionally generated antigen-binding fragments (Fabs) of the antibodies to determine the affinity (*K*_D_) of each for purified pre-F and post-F using biolayer interferometry. The Fab of each antibody in the panel demonstrated an expected binding profile to trimeric pre-F and post-F immobilized on the Octet sensors (Table 1). Antibodies to sites Ø and V exhibited superior neutralization and bound to pre-F but not post-F. Antigenic site I-targeting antibodies, including the canonical site I-binding 131-2A antibody, were the least potent neutralizers and preferentially or exclusively bound post-F. Conversely, antibodies with a reported specificity to site III preferentially or exclusively bound pre-F. There was less conformational bias among antibodies with specificity to sites II and IV, which exhibited varying degrees of neutralization and most bound both pre-F and post-F. Of note, palivizumab and its successor, motavizumab, demonstrated somewhat better affinity for post-F than pre-F. Overall, while our investigational panel of antibodies was limited to several for each antigenic site, the profile of antibodies selected is reflective of the profile established through mapping and functional profiling of the polyclonal response to RSV in humans [21,40].

**Figure 3:**
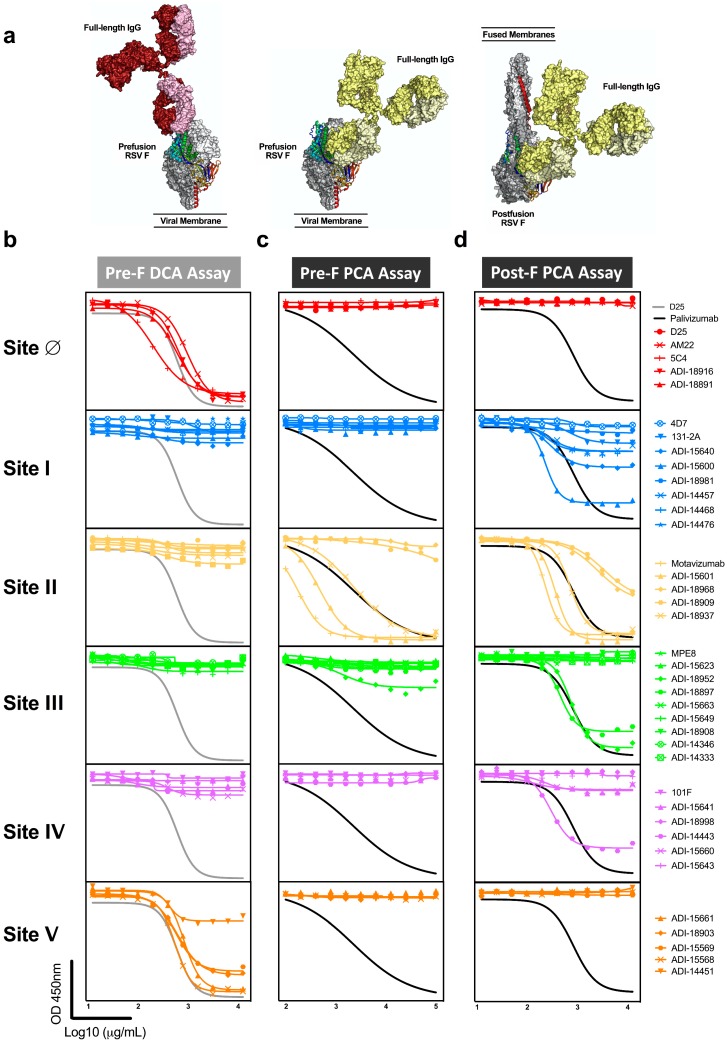
Antibodies targeting adjacent antigenic sites compete with D25 and palivizumab in mAb competition assays. (**a**) One antigen-binding fragment (Fab) of the full-length human IgG1 structure (Protein Data Bank ID: 1IGT) was aligned with the Fab from the complex of pre-F with D25 (red, site Ø), pre-F with motavizumab (yellow, site II), or post-F with motavizumab (yellow, site II) to highlight the relative size of the full-length immunoglobulins (Ig) to pre-F and post-F. (**b**–**d**) A panel of site-specific monoclonal antibodies (detailed in Table 1) was tested for competition in the pre-F DCA (**b**), pre-F PCA (**c**), and post-F PCA (**d**) assay. All of the antibodies in the panel specific to sites Ø and V competed with D25 in the DCA assay (**b**). One site III and multiple site II antibodies competed with palivizumab for binding to pre-F (**c**). Multiple antibodies binding antigenic sites I, II, III, and IV competed with palivizumab for binding to post-F (**d**). Antibodies that exceeded 25% competition of the biotinylated antibody are reported as competing in Table 1.

Due to the relative size of RSV F to monoclonal antibodies, antibodies binding to one site can interfere with antibodies binding to an adjacent site and could contribute to the readout of mAb competing antibody in polyclonal sera (Figure 3a). To test this hypothesis, we queried the panel of 39 mAbs in the pre-F DCA and both pre-F and post-F PCA assays (Figure 3b–d, Table 1). As expected, antibodies specific to antigenic sites I, II, III, and IV did not compete with D25 in the DCA assay, while all antibodies specific for site Ø did compete. In addition, site V-directed antibodies demonstrated substantial competition with D25, demonstrating that antibodies binding at or near the apex can block antibodies to site Ø (Figure 3b). As a result of extensive competition from antibodies targeting neighboring site V, the DCA assay provides a broader readout of antibodies targeting the highly neutralization-sensitive apex of pre-F and is not exclusively a measure of site Ø-specific antibodies.

Next, we assessed the ability of mAbs to block binding of palivizumab to both pre-F and post-F. Despite the presence of sites Ø and V on pre-F, antibodies targeting these sites did not compete with palivizumab in the pre-F PCA assay nor did the antibodies targeting sites I or IV (Figure 3c, Table 1). One of the nine antibodies specific for site III provided competition that exceeded 25% at high concentrations. Not all antibodies with previously mapped specificity to site II were capable of competing with palivizumab in this assay (Figure 3c). Conversely, when post-F was used as a substrate for the PCA assay, all site II-directed antibodies were found to compete with palivizumab. Interestingly, half of the antibodies directed to site I, and some antibodies specific for sites III and IV competed at least 25% of palivizumab binding to post-F but did not compete with palivizumab on the pre-F PCA assay (Figure 3d). These findings demonstrate that the post-F PCA assay not only detects site II-specific antibodies but also antibodies that bind adjacent sites, and that the specificities of antibodies that compete is dependent on the conformation of the F protein.

### 3.4. Vaccine Immunogen Conformation Alters the Relationship of DCA and PCA Readouts to Neutralization

**Figure 4:**
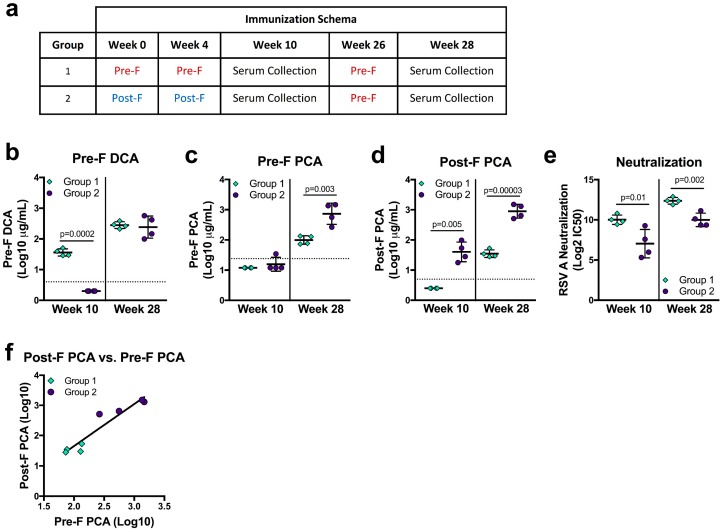
RSV F immune history affects readouts from mAb competition assays. (**a**) Immunization schema and serum collection timepoints for rhesus macaques immunized with 50 µg/animal of RSV F variant immunogen proteins adjuvanted with poly I:C. (**b**–**d**) Serum levels of pre-F DCA (**b**), pre-F PCA (**c**), and post-F PCA (**d**) at weeks 10 and 28. The horizontal dotted lines indicate the limit of quantitation for each assay. (**e**) Neutralization activity of sera collected at weeks 10 and 28. (**f**) Correlation of PCA readouts from group one and group two animals two weeks after the third immunization (*r* = 0.952, *p* = 0.001). All results are the average of technical duplicates performed for each sample.

F-based vaccine candidates currently in clinical trials use pre-F, post-F, or structurally-undefined protein [8,28,48], and the primary application of the DCA and PCA assays are as a readout from clinical studies. To determine how vaccination with pre-F or post-F may influence pre-F DCA, pre-F PCA, and post-F PCA readouts, we analyzed sera from NHP immunized with pre-F or post-F. Indian rhesus macaques were immunized with 50 µg of either pre-F or post-F protein on weeks 0 and 4 and week 10 neutralizing activity was previously reported [28]. Due to overall lower neutralizing antibody in the post-F immunized animals, both groups received 50 µg of pre-F for the third immunization on week 26 (Figure 4a). DCA and PCA levels were measured at study week 10 (six weeks post-second immunization with pre-F or post-F) and at study week 28 (two weeks after a third immunization with pre-F, Figure 4a). At week 10, NHP that received two immunizations with post-F (group 2) had undetectable DCA—since site Ø is not present on post-F—while all animals that received two immunizations with pre-F (group 1) had quantifiable levels (*p* = 0.0002). After a final immunization with pre-F, DCA levels in both groups were not significantly different (Figure 4b). The limit of detection in the pre-F PCA assay precluded quantification for all but one immunized NHP at week 10. Pre-F PCA levels could be quantified at week 28 for both groups and were significantly higher in group two animals (*p* = 0.003, Figure 4c). Despite the lower limit of quantification, week 10 post-F PCA levels could not be quantified for group one NHP and were measurable only in the sera of group two animals (*p* = 0.005). Group two maintained significantly higher post-F PCA levels after the final pre-F immunization was given (*p* = 0.00003, Figure 4d). As a whole, our PCA data suggest that post-F immunogens favor the elicitation of antibodies binding the surfaces shared between pre-F and post-F.

We next asked how mAb competing antibody readouts related to neutralizing activity in the same sera, which was significantly higher in group one than in group two at both week 10 and week 28 (*p* = 0.01 and *p* = 0.002, respectively, Figure 4e). While the trends were similar, DCA was an imperfect predictor of neutralizing activity, as DCA levels in week 28 sera did not corroborate the significant difference in neutralizing activity between groups (Figure 4b,e). Interestingly, pre-F only-immunized animals had undetectable or significantly lower levels of pre-F PCA and post-F PCA despite having significantly higher neutralizing activity (Figure 4c–e). Sera from group two animals offered a similar discordance—significantly lower levels of neutralizing antibody with higher readouts of pre-F and post-F PCA. While neither measure correlated well with neutralization, pre-F PCA and post-F PCA levels correlated well with each other (*r* = 0.952, *p* = 0.001, Figure 4f). While these findings from immunized animals may highlight biases that are not apparent in naturally-infected humans, they demonstrate that immune history may alter the relationship of PCA and DCA to neutralization, cautioning against using these measures as surrogates for neutralization.

## 4. Discussion

Assays continue to be developed to enhance our understanding of the human serological response to RSV F. Given the number of F-based vaccines in the clinical pipeline, it is critical to understand the biology of them and consequently how to interpret the readouts of these assays to better define immune correlates of protection and improve approaches for each of the major vaccine target populations—pregnant women, children, and the elderly. While the antigenic sites on the surface of F are well characterized and the site-specificity of many antibodies has been determined, how steric hindrance complicates measurements of serum antibodies competing with palivizumab and D25 monoclonal antibodies has not been investigated. Here, we optimized assays to quantify antibodies that compete for pre-F binding with the site Ø-directed mAb D25 and compared measurements of antibodies that compete with the site II-directed palivizumab for pre-F and post-F binding. Our comparison of how a panel of monoclonal antibodies competes with D25 and palivizumab in these assays and our demonstration of how these readouts can vary based on immunological history offer caveats and insights for how these readouts should be used in the context of clinical trial analysis.

The threshold for quantifying PCA in the serum of antigen-experienced subjects presents a limitation to understanding how these parameters are impacted by vaccination. Previous studies using post-F as an ELISA substrate for PCA measurements have shown that a significant fraction of acutely-infected and convalescent subjects demonstrate PCA levels below the limit of detection [31,32,49]. Our results using post-F as a substrate are consistent with these findings, as we were only able to quantify post-F PCA in 52% of 58 randomly-selected subjects aged 18–65. Due to the sensitivity of the pre-F PCA assay, the inability to measure this endpoint in the majority of normal subjects may present an obstacle to understanding its utility or could potentially bias data when assessing only subjects with a quantifiable pre-F PCA readout. Post-F PCA and pre-F PCA in subjects where both can be quantitated are correlative, yet even a relatively small panel of monoclonal antibodies clearly delineate how these readouts are impacted by protein conformation and have implications for their utility as a readout of “functional” antibody. Our data show that antibodies to every defined antigenic site on post-F can influence measurements of post-F PCA, and while the pre-F PCA assay provides a more specific readout of site II-directed antibodies, it fails to detect all mAbs previously classified as site II-directed antibodies. Larger studies including both normal and immunized subjects would be necessary to determine how pre-F and post-F PCA results differentially relate to neutralization readouts. Both the conformation of the F protein in each immunogen and the context in which it is presented could affect the relationship of these metrics to neutralization.

The existence of non-neutralizing antibodies that bind site II is well-documented, and it has been cautioned that their contribution to PCA readouts should be considered [21,39]. This variability in the neutralizing potency of site II-directed antibodies alone should elicit hesitancy to consider a PCA value as a measure of “palivizumab-like antibody” or as a surrogate for neutralization. More confounding is our observation that antibodies that bind antigenic site I, preferentially displayed by post-F, could also contribute substantially to this readout. While the prototypic site I-directed antibody 131-2A did not compete in the post-F PCA, four of eight (50%) of our site I-binned antibodies inhibited palivizumab binding by at least 25%. The majority of antibodies targeting site I have little to no neutralizing potential and are unable to bind pre-F [21]. The ability of post-F-containing immunogens to elicit site I-directed antibodies that may contribute to the post-F PCA offers additional warning about the consideration of the PCA as a functional readout. These findings may also account for discordance between measured increases in neutralization versus PCA values following post-F immunization [30]. The undiscriminating nature of the post-F PCA assay makes it difficult to define a level that serves as a clear correlate or surrogate of protection.

Our conclusions contradict a published report claiming that F conformation does not appear to have a measurable impact on PCA assay results [49]. However, these prior studies did not include the use of purified pre-F and instead used cell-based assays where the presence of both pre-F and post-F could preclude the identification of distinctions between assays. The binding of 131-2A antibody to the cell-based protein used suggests this is the case, as 131-2A does not bind to pre-F [49]. Given that the immunological history of antigen-experienced adult subjects is relatively similar, marked by repeated exposure and infection by live RSV decorated with both pre-F and post-F [50,51], it is not entirely surprising that these metrics might correlate. Yet, this baseline profile of F-directed antibodies could change substantially after immunization with antigens with constrained structural topology. Comparing pre-F PCA and post-F PCA results for sera from NHP selectively immunized with different conformations of F serves to demonstrate that the relation of these readouts to neutralization is dependent on immunological history, which may be altered profoundly in a vaccine-experienced population.

Not unexpectedly, all of the mAbs selected to represent the response to antigenic sites Ø and V exhibited the capability to compete with D25. The proximity of these sites on the pre-F apex suggest that the majority of site V antibodies are likely contributors to DCA readouts. In contrast to the antigenic sites shared by both conformations of F, antibodies binding these apex-displayed pre-F-exclusive sites uniformly display favorable neutralization profiles [21]. The DCA assay we developed is an improvement over other described DCA assays in that a µg/mL value could be determined for the majority of human serum samples tested [33,52]. Several studies have demonstrated that pre-F-binding antibodies are the largest contributor to the serological response to F in infected and convalescent humans [21,37,53,54]. The ability to measure improvement in DCA in the majority of clinical trial subjects could make this a critical metric of vaccine response and a more well-defined correlate of protection given the more consistent potency of competing antibodies. Of note, the conformation-dependence of this assay dictates that post-F immunogens or antigens that preferentially-display post-F antigenic sites will inevitably fail to elicit an appreciable boost in DCA, limiting the usefulness of this readout in those studies.

PCA and DCA results are more complex than initially anticipated. Both the conformation of the F protein itself and nature of vaccine immunogens factor into their usefulness as a readout of vaccine responsiveness. In line with other metrics, only empirical testing of larger numbers of samples and improved precision of measurements will reveal their utility. The current vaccine landscape, ripe with pre-F immunogens in a variety of formats, provides several opportunities to do so. Due to the overall variety in F-based immunogens, no mAb competition assay is likely to provide a “one size fits all” metric that replaces a well-standardized neutralization assay. 

## 5. Conclusions

In conclusion, mAb competition assays are routinely used in RSV clinical trial analysis because of their potential to predict protection from infection or disease. We demonstrate that RSV F mAb competition assays are not site-specific, but offer a more general readout of apex- or side- binding antibody levels in sera. Moreover, PCA and DCA readouts are conformation-sensitive, and depending on immunological history, may not correlate with neutralization. Understanding the limitations and complexity of each mAb competition assay is critical to interpreting the usefulness of these readouts. 

## Figures and Tables

**Table 1:** Neutralization and *K*_D_ profile of monoclonal antibodies.

mAb	Reported Antigenic Site	PMID	Neutralization (IC_50_ ng/mL)	Pre-F *K*_D_ (Molar)	Post-F *K*_D_ (Molar)	Pre-F DCA Assay	Pre-F PCA Assay	Post-F PCA Assay
						>25% Competition		
D25	∅	20023635	8.3	1.43 × 10^−11^	*nb*	X		
5C4	∅	23618766	4.3	2.73 × 10^−8^	*nb*	X		
AM22	∅	20023635	5.1	1.92 × 10^−10^	*nb*	X		
ADI−18916	∅	28111638	5.4	1.06 × 10^−9^	*nb*	X		
ADI-18891	∅	28111638	7.4	3.50 × 10^−9^	*nb*	X		
4D7	I	27764150	*nn*	*nb*	2.20 × 10^−9^			
131-2A	I	2459412	*nn*	*nb*	1.69 × 10^−9^			
ADI-15640	I	28111638	180.6	9.53 × 10^−9^	2.15 × 10^−8^			X
ADI-15600	I	28111638	*nn*	*nb*	2.20 × 10^−10^			X
ADI-18981	I	28111638	*nn*	*nb*	3.72 × 10^−10^			
ADI-14457	I	28111638	108.1	1.42 × 10^−9^	4.33 × 10^−9^			X
ADI-14468	I	28111638	115.2	1.18 × 10^−7^	8.99 × 10^−10^			X
ADI-14476	I	28111638	*nn*	*nb*	4.06 × 10^−8^			
Palivizumab	II	9359721	158.6	3.59 × 10^−8^	9.63 × 10^−9^		X	X
Motavizumab	II	17362988	32.9	1.52 × 10^−9^	8.25 × 10^−10^		X	X
ADI-15601	II	28111638	65.6	2.86 × 10^−10^	1.11 × 10^−10^		X	X
ADI-18968	II	28111638	398.9	1.34 × 10^−7^	5.85 × 10^−8^			X
ADI-18909	II	28111638	55.1	3.12 × 10^−7^	2.59 × 10^−7^			X
ADI-18937	II	28111638	14.8	3.20 × 10^−9^	1.27 × 10^−8^		X	X
MPE8	III	23955151	13.2	1.15 × 10^−11^	*nb*			
ADI-15623	III	28111638	18.8	2.95 × 10^−10^	*nb*			
ADI-18952	III	28111638	479.8	3.44 × 10^−8^	1.30 × 10^−9^		X	X
ADI-18897	III	28111638	53.6	*nb*	3.94 × 10^−10^			X
ADI-15663	III	28111638	6.1	1.40 × 0^−9^	*nb*			
ADI-15649	III	28111638	25.5	4.48 × 10^−9^	*nb*			
ADI-18908	III	28111638	24.8	1.27 × 10^−10^	*nb*			
ADI-14346	III	29396163	9.1	5.26 × 10^−10^	*nb*			
ADI-14333	III	29396163	43.4	1.10 × 10^−9^	*nb*			
101F	IV	17872524	94.8	1.45 × 10^−8^	1.49 × 10^−8^			
ADI-15641	IV	28111638	50.0	1.26 × 10^−10^	3.74 × 10^−10^			
ADI-18998	IV	28111638	14.3	3.43 × 10^−10^	*nb*			
ADI-14443	IV	28111638	31.4	2.06 × 10^−10^	3.51 × 10^−10^			X
ADI-15660	IV	28111638	*nn*	1.97 × 10^−8^	8.90 × 10^−11^			
ADI-15643	IV	28111638	24.1	1.37 × 10^−9^	*nb*			
ADI-15661	V	28111638	4.1	3.81 × 10^−8^	*nb*	X		
ADI-18903	V	28111638	20.3	4.46 × 10^−10^	*nb*	X		
ADI-15569	V	28111638	19.3	1.27 × 10^−9^	*nb*	X		
ADI-15568	V	28111638	13.3	2.98 × 10^−10^	*nb*	X		
ADI-14451	V	28111638	12.1	3.58 × 10^−10^	*nb*	X		

*nn* indicates no measurable neutralization of infection. A ratio of *k*_d_/*k*_a_ determined from a 1:1 binding model was used to calculate the *K*_D_. *nb* indicates a non-binder. Antibodies with greater than 25% competition (giving readouts below OD_max_ × 0.75) in the pre-F DCA, pre-F PCA, or post-F PCA assays are indicated with an “X”.
